# Impact of Occupational Cement Dust Exposure on Hematological Health Parameters: A Cross-Sectional Study

**DOI:** 10.7759/cureus.72673

**Published:** 2024-10-30

**Authors:** Rahnuma Ahmad, Md. Ahsanul Haq, Susmita Sinha, Miral Mehta, Santosh Kumar, Mainul Haque, Qazi Shamima Akhter

**Affiliations:** 1 Physiology, Medical College for Women and Hospital, Dhaka, BGD; 2 Bio-Statistics, Infectious Diseases Division, International Centre for Diarrhoeal Disease Research, Bangladesh (icddr,b), Dhaka, BGD; 3 Physiology, Enam Medical College and Hospital, Dhaka, BGD; 4 Pedodontics and Preventive Dentistry, Karnavati School of Dentistry, Karnavati University, Gandhinagar, IND; 5 Periodontology and Implantology, Karnavati School of Dentistry, Karnavati University, Gandhinagar, IND; 6 Pharmacology and Therapeutics, National Defence University of Malaysia, Kuala Lumpur, MYS; 7 Physiology, Dhaka Medical College and Hospital, Dhaka, BGD

**Keywords:** accessible, affordable, contact, cytokines, dust, early change, hemoglobin, inflammation, prevention, toxic metals

## Abstract

Introduction

Contact with the dust of cement consisting of toxic components brings about inflammatory damage (often irreversible) to the body of a human being. The circulatory system exhibits sensitivity to inflammatory changes in the body, and one of the earliest changes may be observed in the blood parameters like mean corpuscular hemoglobin (MCH) and mean corpuscular hemoglobin concentration (MCHC). MCHC and MCH are possibly easily accessible and affordable parameters that can detect harmful changes in the body before any irreversible damage occurs.

Objectives

This research aimed to seek the changes in MCHC and MCH upon occupational contact with the toxic dust of cement.

Methods

The execution of this research was done in the Department of Physiology, Dhaka Medical College, Bangladesh, and a cement plant in Munshiganj, Bangladesh. This research was carried out between September 2017 and August 2018. Individuals (20 to 50 years old, 92 male adults) participated and were grouped into the group with occupational cement dust impact (46 subjects) and the group without occupational dust of cement impact (46 subjects). Data was collected in a pre-designed questionnaire. An independent sample t-test was conducted to analyze statistical and demographic data like body mass index and blood pressure. A multivariate regression model was applied to note the impact of cement dust on the group working in this dusty environment. Again, a multivariate regression model was employed to observe whether the duration of exposure to this dust affected MCHC and MCH. The significance level was demarcated at p < 0.05 Stata-15 (StataCorp LLC, College Station, TX, US) for statistical analysis, and GraphPad Prism v8.3.2 (Insight Venture Management, LLC, New York, NY, US) was employed to present the data graphically when required.

Results

There was a reduction in MCHC by 0.58 g/dL and MCH levels by 0.68 pg in the cement dust-exposed subjects when compared to controls, but not significant (95% CI: -0.93, 2.10; *p *= 0.448 and 95% CI: -0.37, 1.73; *p* = 0.203, respectively). However, MCHC was reduced significantly by 0.51 g/dL (p = 0.011) with the duration of exposure to the dust.

Conclusion

The study showed that MCHC was significantly reduced with the duration of exposure to cement dust in cement plant workers. Such alterations may hamper heme synthesis, hemolysis, and inflammatory changes in the body.

## Introduction

Potential hazards threaten the health of individuals in the place of their work [1]. These hazards include physical hazards (ionizing radiation and noise), ergonomic and psychological hazards (high workload and stress), and chemical hazards (vapors, gases, and dust). When exposed to such agents, the body becomes prone to several occupation-related diseases and complications like cancers, musculoskeletal disorders, and respiratory diseases [1,2]. Industries dealing with silica, lead, and copper are responsible for the emission of toxic substances during the processing or extraction of these elements [3]. Such industries include battery, smelting, mining, ceramics, foundry, glass, and cement [4,5]. Cement is an inseparable component of building infrastructure [6]. The requirement for cement continues to rise as the economy and urbanization increase, particularly in low- to middle-income countries. The ever-growing patterns of urbanization, the need for infrastructure development, and the rise in world population are set to raise the global demand for cement by the year 2050 by about 12% to 23% of that in 2020 [7]. Southeast Asia and Africa are more likely to witness this global rise in demand for cement [8,9].

The composition of cement dust includes aluminum oxide (3%-5%), hexavalent chromium, silicon oxide (17%-25%), calcium oxide (60%-67%), potassium, iron oxide, sulfur, sodium, lead, copper, and magnesium oxide [10]. Dust of cement may enter the body of humans via inhalation, skin, and swallowing [11,12]. There have been reports of aluminum oxide exposure causing peroxidation of lipids in various tissues, leading to renal failure, anemia, and neurotoxicity [13,14]. Chromium in the form of Cr(IV), another toxic component, is reported to be a potent oxidizing agent that causes free radical generation and a rise in inflammation. This leads to deteriorating effects on the liver and respiratory and renal systems [15,16]. Accumulative oxidative damage to components of cells and alterations in functions of cells have been attributed to continuous reactive oxygen species (ROS) efflux [17,18].

Excessive exposure to lead is related to raised blood pressure, anemia, infertility, and damaging effects on the nervous and renal systems [5,19]. Although the carcinogenic impact of lead is unclear, the International Agency for Research on Cancer (IARC) considers lead a possible human carcinogen [5,20]. Another effect of lead is shortening red blood cells' (RBCs’) lifespan, which leads to reticulocytosis and anemia [21,22]. Chronic exposure to harmful components such as lead hinders the body from producing hemoglobin by disrupting the pathway's heme synthesis enzymes, which raises the risk of developing anemia [23]. A deficiency of iron that occurs due to lead absorption may also cause anemia [24].

Occupational exposure to silica, a significant constituent of this dust, is responsible for the development of an irreversible disease of the lung known as silicosis. Due to the engulfment of silica (entering via inhalation in the respiratory tract), the subject develops nodular lesions, lung fibrosis, and inflammation. Silicosis may lead to respiratory tract infection, pneumothorax, and respiratory failure [25]. In China alone, over 230,000 workers are exposed directly to respirable crystalline silica, and silicosis is one of the most prevalent diseases in the country [26]. Silicosis cases are also increasing in glass, nanomaterial, and jewelry-making industries [27,28]. Globally, respirable crystalline silica has become a threat to the development of silicosis in millions of workers. However, silicosis diagnosis depends on abnormalities of pulmonary functions and irreversible alterations observed radiographically. Early detection by use of biomarkers is a necessity for the health of these individuals. Even though various biomarkers have been noted for silicosis in occupationally exposed subjects and animal experiments, these lack diagnostic specificity and sensitivity, require high technological support, and are unsuitable for conducting in a large population. Routine blood parameters are probably a non-invasive marker for the routine assessment of the health of these workers [29-36].

Dust inhalation is also related to another occupational disease called pneumoconiosis. This occupational disease is responsible for high morbidity. Like silicosis, pneumoconiosis is evaluated clinically through the observation of radiological alterations. Early diagnosis is of much benefit to preventing extensive damage to workers' lungs from dust exposure [37]. Pneumoconiosis is one of the irreversible damages that may occur due to the inhalation of cement dust and is an outcome of inflammatory changes in the respiratory system. Studies have been conducted to find easily accessible biomarkers to determine inflammatory changes early in the respiratory system, possibly leading to pneumoconiosis [38,39]. The hemoglobin concentration in each liter of blood is represented by mean corpuscular hemoglobin concentration (MCHC) [38-40]. Over the years, studies have emerged concerning the clinical application of MCHC. Several research works have noted an association between MCHC and diseases like hepatorenal syndrome, acute myocardial infarction, and depression [41-43]. Relationships have also been reported between MCHC and lung disease [44,45]. The poor prognosis of lung carcinoma has been associated with lowered MCHC [45]. A decrease in MCHC has been linked to a rise in mortality in individuals suffering from acute pulmonary embolism [44]. Hemoglobin-related parameters have been reported to vary in recent research in pneumoconiosis patients [46].

The human hematopoietic system is a good indicator in toxicological research since it exhibits extreme sensitivity to alterations in the surrounding environment in which increasing metabolic demands cause rapid production and breakdown of cells [47]. Previous studies have linked a decrease in MCHC to lung diseases [44,45]. A reduction in MCHC has been noted in the advanced stages of pneumoconiosis [38]. Studies have observed alterations in hematological parameters on cement dust exposure [47-52]. However, the modifications in MCHC and mean corpuscular hemoglobin (MCH) of workers having contact with cement dust remain unclear. This research aims to note the MCH and MCHC alterations in those exposed to dust during cement production. Since toxic cement components promote oxidative stress and inflammation and reduce hemoglobin synthesis, they may encourage anemia [52-56]. Also, changes in these parameters may act as an early detection tool that is easy to conduct and affordable for assessing harmful changes in these workers.

Objective of the study

The research objective is to note the effect occupational exposure to cement dust has on the hematological parameters of MCHC and MCH in a Bangladeshi cement factory.

Problem statement of this study

Pneumoconiosis is one of the irreversible damages that may occur due to the inhalation of cement dust and is an outcome of inflammatory changes in the respiratory system. Studies have been carried out to find easily accessible biomarkers to determine inflammatory changes early in the respiratory system, which possibly lead to pneumoconiosis [15,24,27-29]. Being in contact with components like silica, hexavalent chromium, and lead may bring about oxidative stress [29-31]. The hematopoietic system may be impacted early in inflammatory damage [57-59]. Components of cement like lead may hamper the synthesis of hemoglobin and absorption of iron, contributing to the reduction in hemoglobin synthesis and leading to the development of anemia [60,61]. Changes in MCHC and MCH would suggest morphological changes in RBC hinting toward anemic changes in the circulation [62,63]. These biomarkers may, thus, be used on a broad scale to determine the toxic effect of cement dust on impoverished workers who often can neither afford nor have access to high-end scientific laboratory-based marker tests [38].

## Materials and methods

Study design

This research was a cross-sectional study.

Study place and period

The research was conducted at Dhaka Medical College, Bangladesh, and at a cement manufacturing plant in Munshiganj between September 2017 and August 2018.

Study population

The population of this research work was divided into a study group (participants working in the cement plant of Munshiganj, having exposure to dust that is released while manufacturing cement) and a control group (recruits living around the city of Dhaka, with no known encounter of the dust of cement plant). In line with a study performed earlier [52], recruits from sections like crushing, bagging, loading, and milling parts of the cement plant were chosen since these parts of the plant have the most dust release. As for the control group, those chosen had no history of traveling to areas with cement dust or other toxic dust exposure within six months before this study. Their occupation and residence were taken to ensure they were further free from such dust contact.

Selection criteria

The recruit's selection depended on the following inclusion and exclusion criteria.

Inclusion Criteria

The inclusion criteria include individuals in the age group of 20 to 50 years (inclusion criteria for both the study and control groups) and subjects with no known chronic or acute disease (criteria of inclusion into the study for both the study and control groups). When choosing the study group, those who had contact with cement dust while working in a cement plant for two or more years were included. This was in line with research done previously to assess the effect of cement toxic dust on the cellular component of human blood since chronic inflammation may produce its effects slowly, taking years to cause changes in the body [58].

Exclusion Criteria

The exclusion criteria (applied for both the study and control groups) include subjects having a history of allergies and receiving chemotherapy, anticoagulants, blood transfusion, or iron therapy within three months before the performance of the research. The research excluded those with a history of suffering from disorders or diseases related to the blood, kidneys, liver, respiratory system, or gall bladder. Subjects with a history of acute or chronic infections and malignancies were also excluded.

Sampling technique

The sampling technique applied for this research was non-randomized purposive sampling.

Sample collection

Three milliliters of blood from each participant was taken and mixed with anticoagulant ethylenediaminetetraacetic acid (EDTA) [64]. Blood examination and MCHC and MCH tests were performed in the Department of Laboratory Medicine (Dhaka Medical College) with an automated hematology analyzer (Horiba Pentra DX (Horiba ABX SAS, Montpellier, France)). Validation of this analyzer was assessed by studies done by Hur et al. and Kim et al. [65,66]. Hur et al. noted that its specificity and sensitivity were 93.7% and 89.8%, respectively [65]. Kim et al. observed that the Pentra DX hematology analyzer showed better-flagging performance and better correlation with manual analysis when compared to the Sysmex XE-2100 (Sysmex, Kobe, Japan) hematology analyzer [66].

Data collection

After ensuring that the inclusion criteria were met, the study population answered a structured questionnaire adapted from the Occupational Safety and Health Administration's standard questionnaire [67,68] and modified Kuppuswamy socio-economic scale [69]. The questionnaire was completed through a face-to-face interview. The first segment of the questionnaire for both the study and control group included queries regarding age, locality, and sex. Also included were queries about alcohol intake, drugs consumed, medical conditions of the participants, and medical conditions like bronchial asthma, hypertension, and diabetes mellitus existing within their family. To ensure that the control group was free from exposure to cement dust, their history of travel, residence and work location, and occupation were taken. Table 1 exhibits the drugs regarding which information was taken and Table 2 presents the medical condition history of the subjects to ensure that the participants were not taking any medication therapy or suffering from any chronic disease that may bring changes to the hematological system.

**Table 1:** The history of drug consumed to determine inclusion into the study; exhibiting the drug history that influenced the study subject selection.

Drugs that participants were inquired about
Steroids
Drugs that suppress allergy
Drugs taken to control diabetes mellitus
Drugs taken to control hypertension
Chemotherapy
Therapy with iron
Drugs that reduce blood coagulation
Drugs used to dilate bronchi
Supplementation with nutrients like vitamins

**Table 2:** The medical history taken to determine inclusion into the study subjects; exhibiting the medical history that influenced the study subject selection.

Medical conditions that participants were inquired about
If subjects are suffering from any allergies
If they are having bronchial asthma
If the subjects are diagnosed with diabetes mellitus
If they are suffering from chronic obstructive pulmonary disease (COPD)
If they have any malignancies
If they have been diagnosed with hypertension
If the subjects suffer from any acute or chronic infections (acute/chronic)
If the subjects have any kidney diseases
If they have any liver hepatic diseases
If the subjects suffer from a deficiency of iron
If the subjects have taken any blood transfusion within three months before becoming a part of the study
If the participants have undergone any surgeries before the study: surgery done on them recently
If the participants suffer from thalassemia

Workers of the cement plant (study group) answered the questionnaire's next segment regarding the duration of their exposure to cement dust and the section of the cement plant they worked in. Questions were also made regarding their knowledge of personal protective equipment (PPE), the significance of using PPE, and the health hazards that can develop when exposed to this dust. The last segment of the questionnaire was dedicated to collecting anthropometric measurements like weight, height, and body mass index (BMI), as well as systemic and general physical examination findings like recording blood pressure and pulse values. The study group worked in plant sections with maximum dust emission, such as sections for milling, crushing, bagging, and loading, like previous research [52].

Ethical approval

This research work's ethical approval was obtained from the Research Review and Ethical Review Committee (Dhaka Medical College, Dhaka, Bangladesh) on 02.01.2018 with reference number MEUDMC/ECC/2018/06. Details regarding the research and procedure to be performed were explained to the study participants, and following this, written consent was obtained from each of them.

Research work's impact

This research work may aid in finding a blood parameter for detecting inflammation in the body due to being exposed to dust or cement early, which is inexpensive, accessible, and easy to test. Measures may be taken early to prevent further damage to various body organs due to inflammatory changes. An affordable test may be easily performed for workers living in poverty on a large scale. The policymakers and owners of companies would be motivated to include, as part of the routine health investigations, such low-cost parameters to determine any damages to workers' health.

Statistical analysis plan

**Figure 1:**
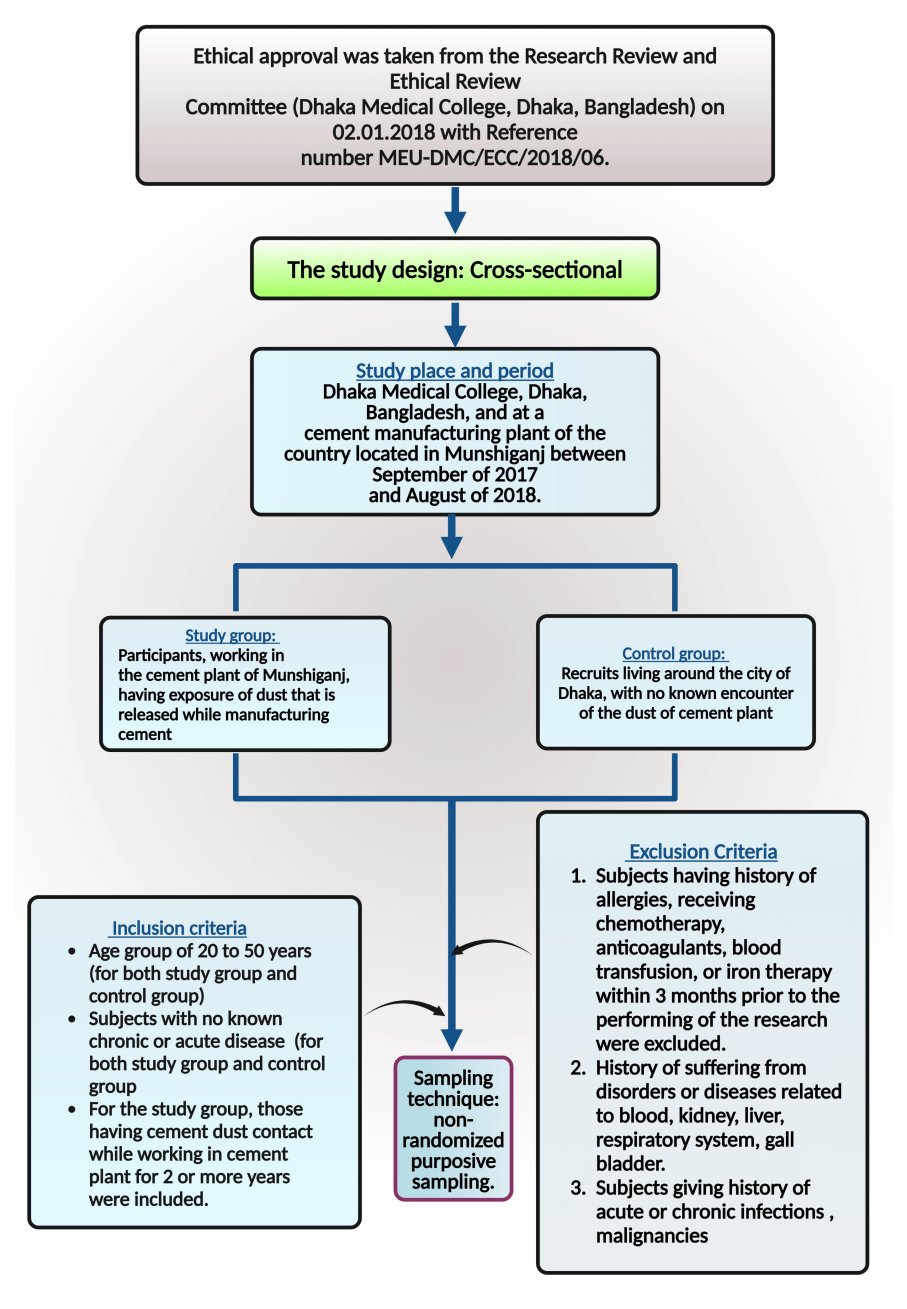
Illustration of the materials and methods employed in this study. The premium version of BioRender (https://biorender.com/) was used to draw this figure, which was accessed on September 17, 2024, with license number ZS27BDWWI5 [70]. Image credit: Susmita Sinha.

Demographic characteristics were determined by employing descriptive analysis. Cross-tabulation was performed for categorical variables like BMI, and an independent sample t-test was done for continuous variables. Multivariate regression analysis was done to see the exposure to dust effect in a study group in comparison to the participants of the control group. Adjustment by age and BMI (category) was done for the regression model. A multiple regression model (adjusted for age and BMI) was also used to assess the impact of exposure duration over the years in the study group. p-value < 0.05 was considered to be significant in this analysis. Stata-15 (StataCorp LLC, College Station, TX, US) was used for the statistical analysis, and GraphPad Prism v8.3.2 (Insight Venture Management, LLC, New York, NY, US) was used for the graphical presentation. Figure 1 illustrates the materials and method of this research work.

## Results

The information on the demography of the participants is shown in Table 3. The total number of participants in the study group was 46, and the same number of participants was enrolled in the control group. The mean age was comparable in the study group and control group, which was 33.2 (±8.37) and 33.5 (±7.96) years, respectively. BMI category distribution revealed that the study group participants with normal BMI were 76.1% while 23.9% had a BMI above average. The BMI of the control group showed that 63.0% of the participants had a BMI within the normal range, while 37.0% had a BMI above the normal range. Slight variations were noted in the blood pressure readings between the two groups. Here, the study group participants had a mean systolic blood pressure of 117.7 ± 15.2 mmHg and diastolic blood pressure of 71.7 ± 9.61 mmHg. In comparison, for the control group, the systolic blood pressure was 113.0 ± 12.2 mmHg, and the diastolic blood pressure of the control group was 70.9 ± 9.21 mmHg. Those enrolled in the study group (cement plant workers) had been exposed to cement dust for an average of 7.17 years.

**Table 3:** Demographic information of the participants enrolled. The presentation of the data was done as the mean ± SD or number with percentage in the parenthesis. The p-value was estimated using a chi square for 2 x 2 contingency observation, and an independent sample test was performed for continuous observation. SBP: systolic blood pressure; DBP: diastolic blood pressure; BMI: body mass index.

Variables	Study group (n)	Control group (n)	p-value
BMI (kg/m^2^)	-	-	-
Normal	35 (76.1%)	29 (63.0%)	0.174
Above normal	11 (23.9%)	17 (37.0%)	
Age	33.2 (±8.37)	33.5 (±7.96)	0.839
DBP (mmHg)	71.7 (±9.61)	70.9 (±9.21)	0.659
SBP (mmHg)	117.7 (±15.2)	113.0 (±12.2)	0.108
Exposure duration (years)	7.17 (±2.82)	-	-

**Figure 2:**
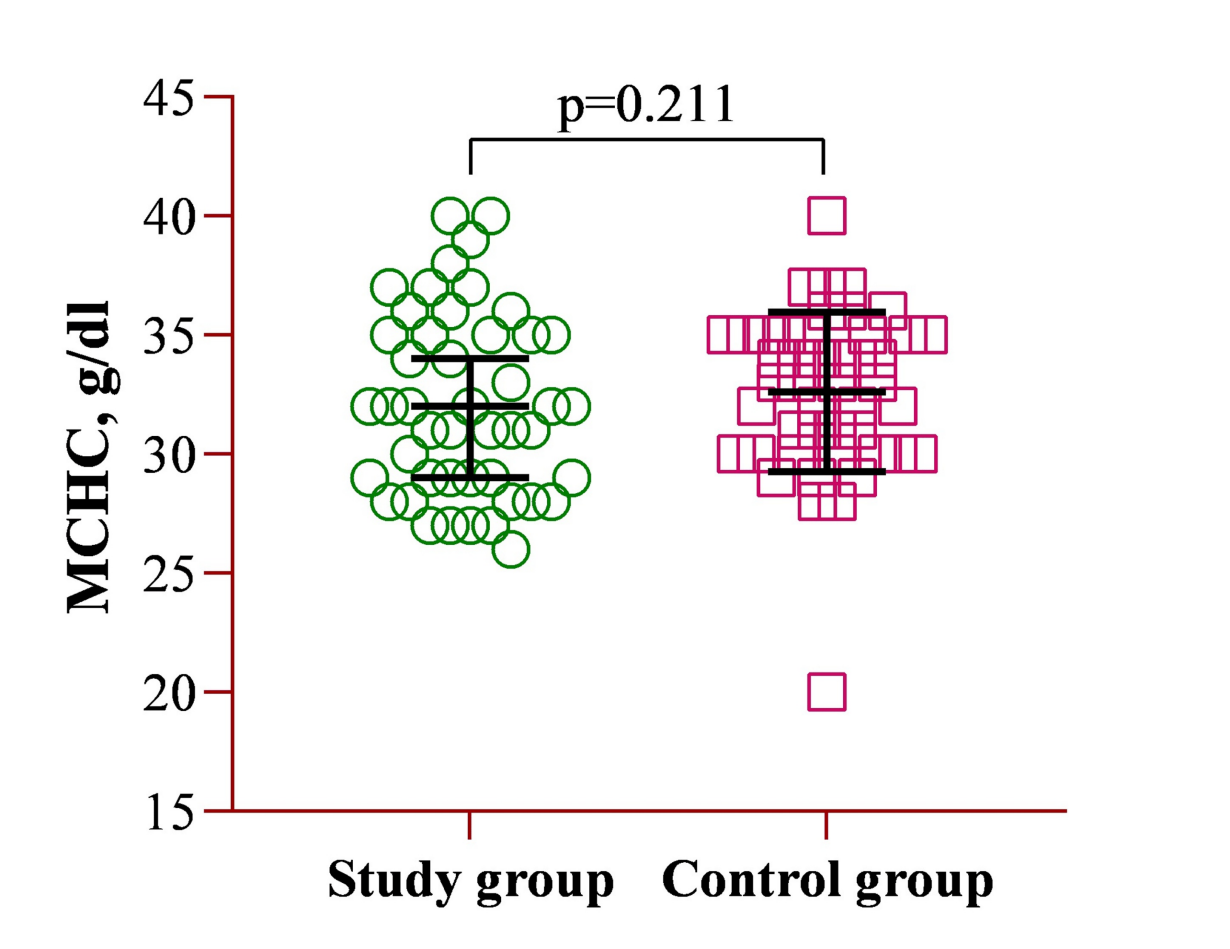
Illustration of the CBC parameter of MCHC. MCHC: mean corpuscular hemoglobin concentration; CBC: complete blood count.

The mean MCHC concentration for the study group was 32.1 ± 3.85 g/dL, while for the control group, it was 32.6 ± 3.35 g/dL. A multivariate regression analysis revealed no significant difference between the two groups (Figure 2).

**Figure 3:**
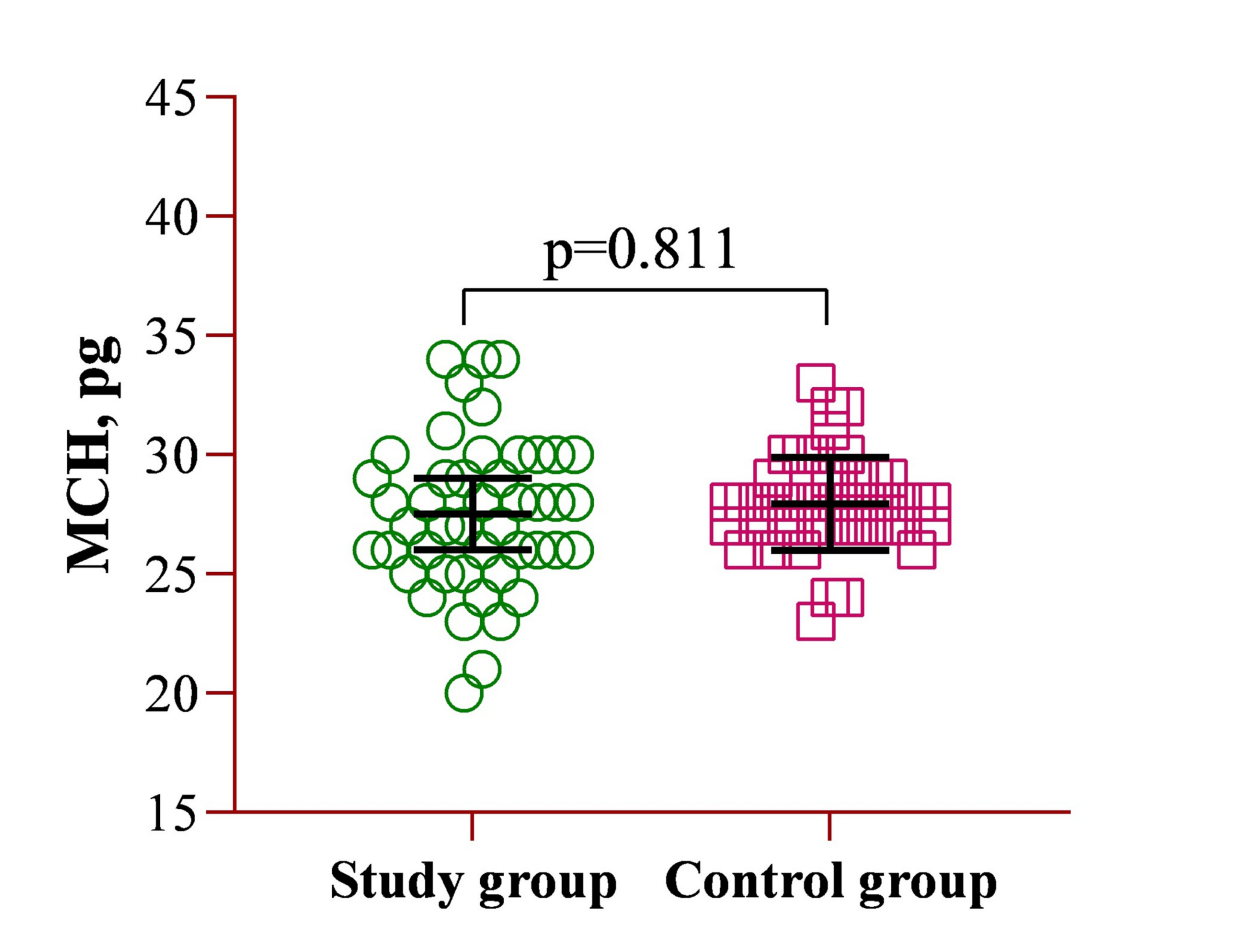
Illustration of the CBC parameter of MCH. MCH: mean corpuscular hemoglobin; CBC: complete blood count.

Similarly, comparing the mean MCH values between the study group (27.5 ± 3.19 pg) and the control group (27.9 ± 1.95 pg) showed no statistically significant difference. Despite slight variations in the mean values, the results suggest that MCH levels were comparable between both groups, as confirmed by the statistical analysis (Figure 3).

**Figure 4:**
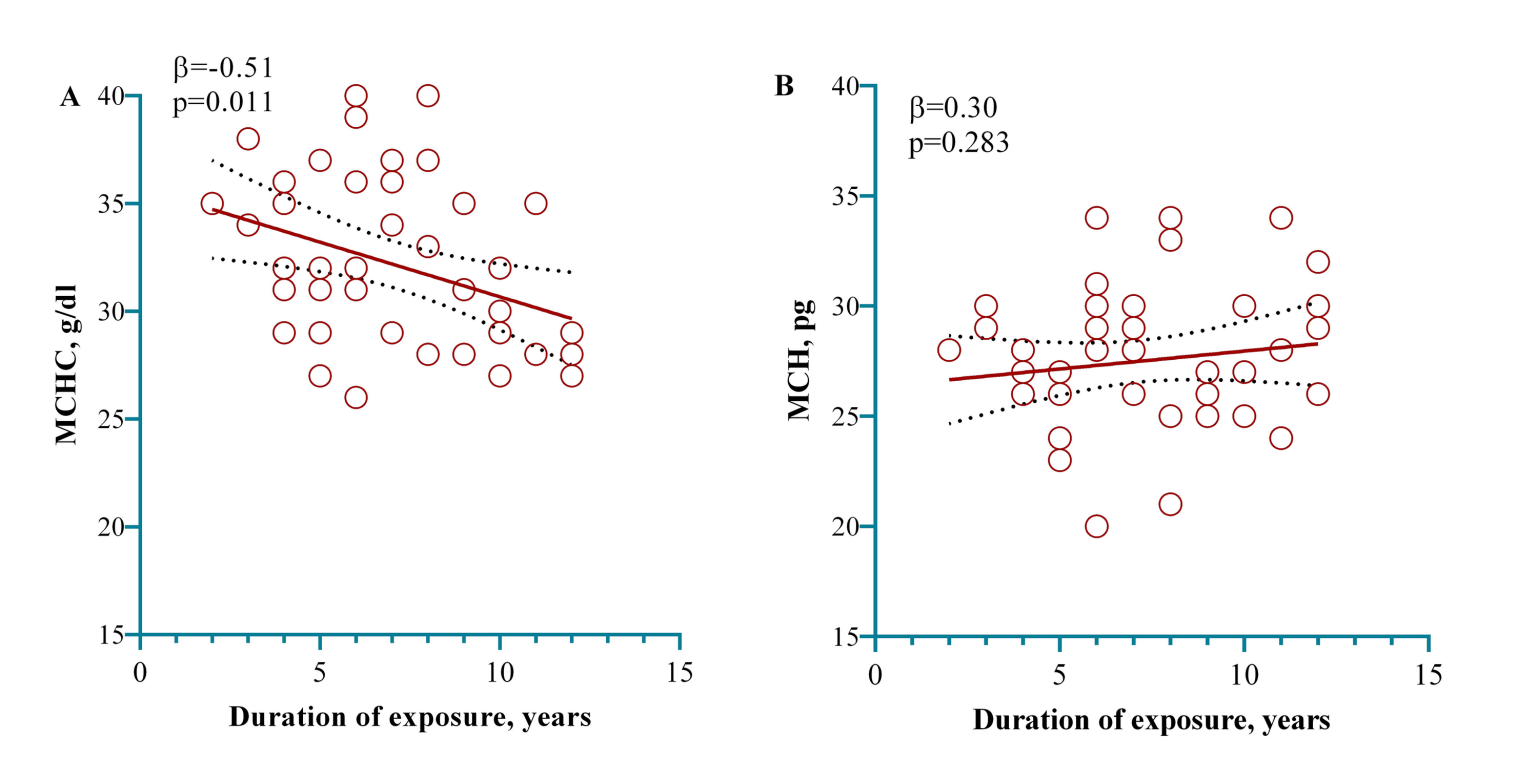
A model showing that one year of exposure working in a cement factory significantly decreased the MCHC. The relationship (linear) between contact with the dust of cement and MCHC (A) and MCH (B) was examined here. The indication of relationship linearity is shown employing the dots in red, in which individual observations are represented by the dots in red. Calculating the p-value was done using a multivariate regression model adjusting for age and BMI (categorical) in the regression model. MCHC: mean corpuscular hemoglobin concentration; MCH: mean corpuscular hemoglobin; BMI: body mass index.

A multivariate regression model was employed to see the association with the dust of cement contact duration within the study group participants. The model showed that contact with cement dust occupationally, for one year in a plant manufacturing cement, exhibited a significant decrease in MCHC by 0.51 g/dL (p = 0.011), as shown in Figure 4.

## Discussion

This research study examined the impact of occupational contact with cement dust on blood parameters MCHC and MCH. Dust exposure indicates possible bodily inflammatory alterations due to contact with the dust. The significant decrease in MCHC with duration was independent of socio-demographic characteristics [71].

Cement dust contact harms human health, and the damages are often irreversible. A study by Shanshal and Al-Qazaz noted abnormal spirometry readings and a significant lowering of the function of the lung (p < 0.001) in the case of occupational contact with the dust of cement in comparison to control subjects [72]. Similar outcomes of reduced lung function, particularly abnormal peak expiratory flow (PEF), were observed by Omidianidost et al. in cement plant workers [73]. Grain warehouse workers in Costa Rica were reported to suffer from raised concentrations of respirable dust higher than the standard cut-off mark [74]. Impairment of renal function (p < 0.05) was noted by Bama et al. in Indian workers in the construction industry having been in contact with rock dust [75]. Dust emitted from the welding industry contains high volumes of heavy metals like manganese, nickel, hexavalent chromium, and aluminum [76,77]. Personnel working in rice mills are also at risk of exposure to a speck of rice dust, which may contain microbes, endotoxins, elements, and spores [78]. Therefore, respiratory tract diseases and poor lung function are common among rice mill workers primarily present in Bangladesh and India compared to those not in contact with such dust [79,80].

Dust like that in the streets also poses a threat to the health of human beings because of bioaccumulation, toxicity, and persistence. City street dust consists of zinc, chromium, copper, cadmium, arsenic, nickel, manganese, lead, and mercury above the safety levels marked by world soil background values. The health risk levels of heavy metal of street dust determined by the United States of America Environmental Protection Agency's health risk evaluation model were 5.71 × 10^-3^ in adults and 2.57 × 10^-2^ in children [81,82]. Barium, lead, and copper levels were noted by Liu et al. to be high in road dust [83]. The air surrounding mercury mines and compact fluorescent lamps were heavily polluted with mercury [84,85]. Mercury has been suggested to be a possible carcinogen in research [86]. Long-term and transient contact with chromium-containing dust has been linked to respiratory tract carcinoma and respiratory function deterioration [87].

**Figure 5:**
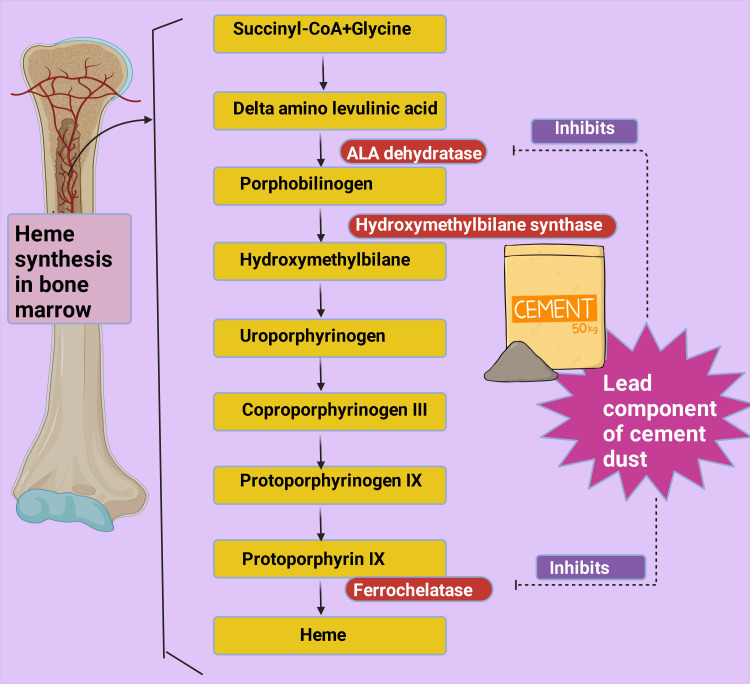
Depiction of the heme production steps in which enzymes like ferrochelatase and delta-aminolevulinic acid dehydratase (ALA dehydratase) are inhibited by lead, which blocks heme formation. The premium version of BioRender (https://biorender.com/) was used to draw this figure, which was accessed on September 14, 2024, with agreement license number ND27B0I06W [70]. Image credit: Rahnuma Ahmad.

Several mechanisms may be at play that led to the study's findings. One possible pathophysiology may be due to anemia resulting from chronic inflammation. Toxic dust components may trigger inflammatory cytokines like interleukins and tumor necrosis factor-α (TNF-α), which have been noted to rise in cement dust exposure [88]. A study reported that patients with lung conditions like pneumoconiosis, which is aggravated by occupational dust inhalation, have higher levels of cytokines like TNF-α and interleukin-8 when compared to control subjects [89]; another study found raised levels of TNF-α and interleukin-1 in individuals with pneumoconiosis [90]. Anemia in inflammation may be attributed to developing resistance to erythropoietin [91,92]. In chronic inflammation, cytokines like TNF-α and interleukin-1 hampered erythropoiesis [93]. Erythropoietin resistance may result in a decrease in MCHC [38]. Lead is a component of cement dust, which has a deteriorating effect on heme synthesis by blocking the enzymes of the heme synthesis path and increasing the chance of anemia development [94]. Enzymes like ferrochelatase and delta-aminolevulinic acid dehydratase (ALA dehydratase) are inhibited by lead, which in turn blocks heme formation (Figure 5) [95-97]. Erythropoietin formation is also disrupted by this heavy metal, which prevents the maturation of RBCs [98,99]. Pyrimidine 5'-nucleotidase deficiency occurs in the presence of lead, resulting in hemolysis [100,101]. A reduced hemoglobin level was reported by Ukaejiofo et al. (p < 0.0001) in workers who handle lead occupationally in comparison to controls [102].

A study done by Kargar-Shouroki et al. on workers of a battery-producing factory in Iran noted a significant reduction in MCH and MCHC in those exposed to lead at work [103]. Absorption of lead leads to iron deficiency [24]. Studies have noted that chronic exposure to harmful components such as lead hinders the body from producing hemoglobin by disrupting the enzymes of the pathway for heme synthesis, which raises the risk of developing anemia [92]. Deficiency of iron that occurs due to lead absorption may also cause anemia [24]. Iron deficiency is often found in individuals with chronic inflammation [104]. A deficiency of iron can reduce MCHC significantly. The research observed an association between deficiency of iron and MCHC [105].

Cadmium and lead exposure may raise lead bioavailability, affecting the enzymes of heme formation steps [56,93]. Both cadmium and lead aggravate inflammation as they compete with intracellular iron, increasing the quantity of free iron that is not bound. This may promote hydrogen peroxide, superoxide, or metal systems and form ROS intermediates [106-108].

**Figure 6:**
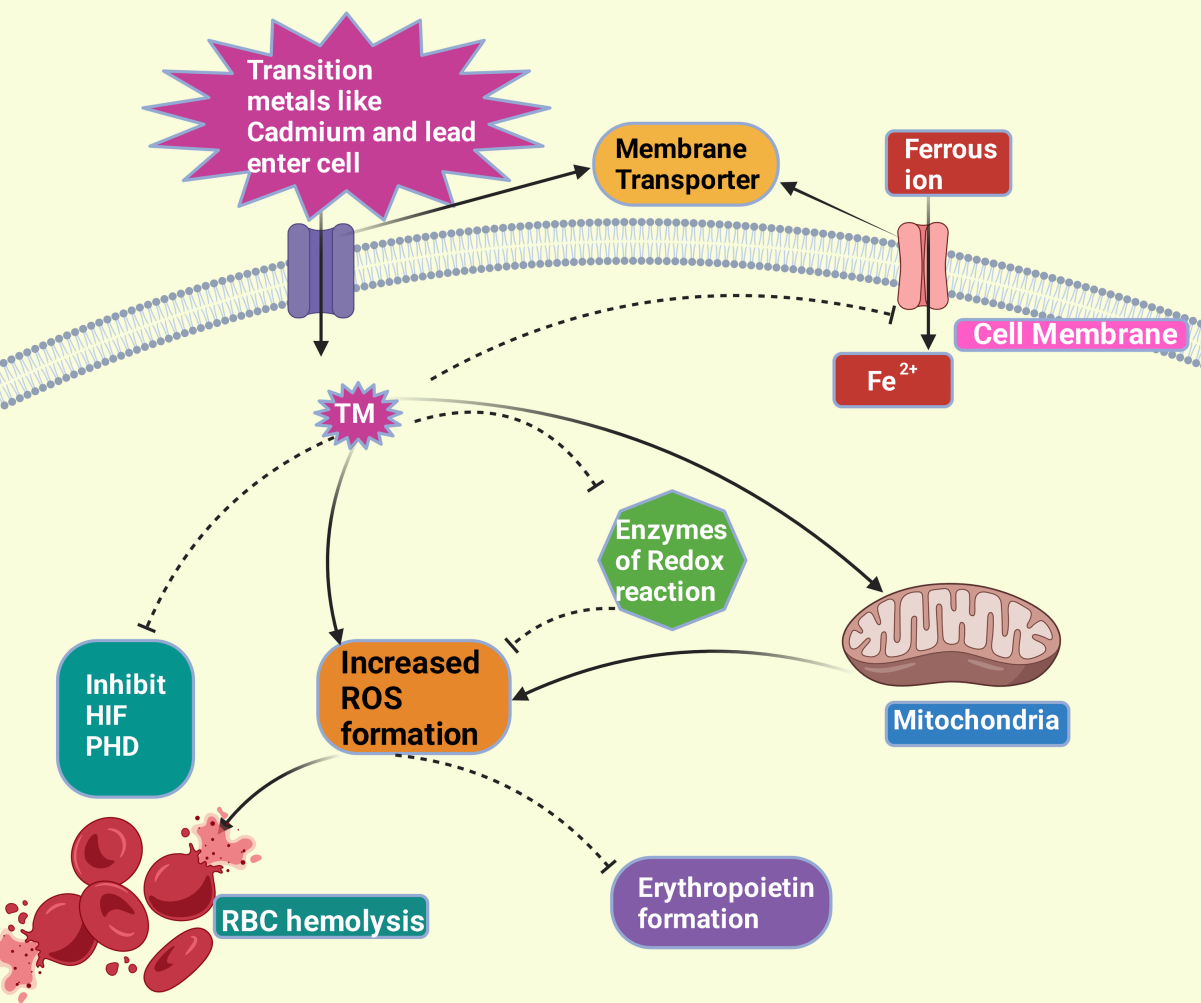
This figure explains how the entry of transition metal promotes reactive oxygen species (ROS) production either by inhibiting the scavengers of ROS or employing their redox reactivity or via disruption of the electron transport chain of the mitochondria; inhibits entry of iron into a cell; inhibits HIF and PHD. The ROS, in turn, inhibits erythropoietin formation and causes red blood cell hemolysis. This figure was drawn using the premium version of BioRender (https://biorender.com/), accessed on September 14, 2024, with agreement license number NX27B0RZ5D [70]. PHD: prolyl hydroxylase domain enzymes; HIF: hypoxia-inducible factor; Fe^2+^: ferrous ion; TM: transition metal; RBC: red blood cell. Image credit: Rahnuma Ahmad.

An animal study on rodents showed that cadmium exposure resulted in hemolysis [109]. Transient metals like cadmium inhibit hypoxia-inducible factor-1 and erythropoietin induction by generating intermediates of ROS, resulting in oxidative hemolysis (Figure 6) [106,110-112]. Heavy metals in cement dust may release highly reactive species, causing DNA damage, protein depletion, and lipid peroxidation. These metals can displace metals that are endogenous from carrier protein ligands. Hydroxyl ions may be produced by the Fenton reaction, giving rise to nitrite [113]. Copper(II), zinc(II), and manganese(II) cause redox reactions and aggravate cytotoxicity [114].

Several studies agree with our research. The hematocrit and hemoglobin levels have been reported to decrease significantly upon contact with dust or cement by Mojiminiyi et al. [48]. Another study on MCHC levels in pneumoconiosis patients noted a significant decrease in MCHC with the advancing stage of pneumoconiosis. They suggested that with the progress of inflammation and damage, there was a decrease in MCHC. Our study also found a reduction in MCH levels (although not significant) upon cement dust contact when compared to controls, and an insignificant negative association was also noted between MCH and duration of contact with the dust. Jacob et al. found that MCH was reduced significantly in occupational contact with this cement dust [115]. They attributed the change to a hemolytic reaction aggravated by chromium present in cement dust along with nickel, copper, and lead. They also mentioned that cement dust bioaccumulation has been reported to cause osteonecrosis and cortex thinning in the bones of animals, reducing epiphysis cartilage gradually [116,117]. This may also occur in humans and reduce absorbed dietary iron storage [115]. However, Mandal and Suva observed a rise in MCH and MCHC [118]. Such variation may be due to differences in the occupational group, extent and duration of contact with the dust of cement, and nutritional status of the workers. Table 4 gives the key findings of this study.

**Table 4:** The key findings of this narrative review. MCH: mean corpuscular hemoglobin; MCHC: mean corpuscular hemoglobin concentration.

Key findings of this paper
Dust of cement may have damaging and often irreversible negative impacts on human health
Several transition metals are components of this dust including lead, hexavalent chromium, and cadmium
Such metals promote inflammation, inhibit heme synthesis, compete with iron absorption, and cause the formation of reactive oxygen species
This study observed a reduction in MCH and MCHC in subjects with cement dust contact compared to the control subjects. Moreover, a significant decrease in MCHC was noted with increasing duration of cement dust contact in those occupationally exposed to this dust
Such alterations in MCHC may suggest the impact of inflammatory changes on the hematological system and may be used to detect early changes due to inflammation in the human body
Checking parameters like MCH and MCHC is easy to perform, affordable, and accessible and thus may be included as part of the routine physical examination of the occupational dust of cement-exposed workers
Early detection of inflammation is necessary to prevent irreversible damage to the health of workers, and in addition, awareness needs to be developed among these workers concerning the harmful effects of being in contact with the dust of cement, and they must be encouraged to use personal protective gear while working in this dusty environment

Limitations of the study

This research has certain limitations. The cause-effect relationship could not be obtained since the study was cross-sectional. Due to financial, time, and technical constraints, we could not research the association of each heavy metal to MCH and MCHC levels in the participants. Due to the mentioned constraints, we could not find the pathophysiology, which was dose- and time-dependent. Our research did not conduct tests to obtain the study subjects' erythropoietin, iron, and ferritin levels. Therefore, the direct link connecting to the reduction in MCHC could not be assessed. Due to time and financial limitations, the research was executed on those working in a single plant manufacturing cement.

Recommendation for future research

The study involved the workers of one factory producing cement in the country. A wide-scale, long-term, and follow-up study needs to be performed to recognize the connection between cement dust contact and MCHC and MCH. The association between each of the constituents of cement dust, MCHC, and MCH should also be studied. Policymakers and cement company owners should consider parameters like MCHC alterations, particularly those with over one year of cement dust exposure. They should encourage workers to take this accessible and affordable test for early inflammatory change detection in the body when working in a dusty environment for a prolonged time. Factory owners and those who make policies should also educate the workers regarding the importance of using PPE like masks, helmets, and gloves to lessen dust exposure [119]. Monitoring the dust emitted by the cement plants and the emission level should be limited as much as possible [120].

## Conclusions

Disruption of homeostasis within the body may occur due to occupational contact with toxic dust emitted during cement production through inflammatory changes and oxidative stress. Exposure to metals like lead and chromium (components of cement dust) may inhibit enzymes of heme synthesis, compete with iron absorption, and promote inflammation and production of ROS. Inflammatory mediators like TNF-α can hamper erythropoiesis. The ROS also may cause damage to DNA, depletion of protein, and peroxidation of lipids. These metals can displace metals that are endogenous from carrier protein ligands. Hydroxyl ions may be produced by the Fenton reaction, giving rise to nitrite. This study observed a significant decrease in MCHC with the duration of exposure to the dust of cement, which may indicate a change in the hematological system related to hemoglobin. The inflammatory mediators may bring about organ damage. Several parameters for detecting inflammation in the body require high-tech laboratories and are expensive to perform. Determination of MCHC is an easily performable, affordable, and accessible test that can be included in routine health checkups of workers who encounter this dust. Any changes should be observed, and necessary steps should be taken to safeguard their health so that further inflammatory damage does not occur and these workers can remain in the workforce for a long time in good health.
